# 2,2′-[(*E*)-3-(4-Nitro­phen­yl)prop-2-ene-1,1-di­yl]bis­(3-hy­droxy-5,5-dimethyl­cyclo­hex-2-en-1-one)

**DOI:** 10.1107/S1600536812032242

**Published:** 2012-07-21

**Authors:** Joo Hwan Cha, Yong Seo Cho, Jae Kyun Lee, Junghwan Park, Hiroyasu Sato

**Affiliations:** a Advanced Analysis Center, Korea Institute of Science & Technology, Hwarangro 14-gil, Seongbuk-gu, Seoul 136-791, Republic of Korea; b Center for Neuro-Medicine, Korea Institute of Science & Technology, Hwarangro 14-gil, Seongbuk-gu, Seoul 136-791, Republic of Korea; c Corporate R&D Center, Duksan Hi-Metal Co. Ltd., Cheonan-si 331-821, Republic of Korea; d Application Laboratory, Rigaku Corporation, 3-9-12 Matsubara-cho, Akishima-shi, Tokyo 196-8666, Japan

## Abstract

In the title compound, C_25_H_29_NO_6_, each of the cyclo­hexenone rings adopts a half-chair conformation. The hy­droxy and carbonyl O atoms face each other and are oriented to allow for the formation of two intra­molecular O—H⋯O hydrogen bonds. In the crystal, weak C—H⋯O hydrogen bonds are formed between molecules, generating a two-dimensional supramolecular structure.

## Related literature

For related structures, see: Cha *et al.* (2011 ▶, 2012 ▶); Zhu *et al.* (2011 ▶).
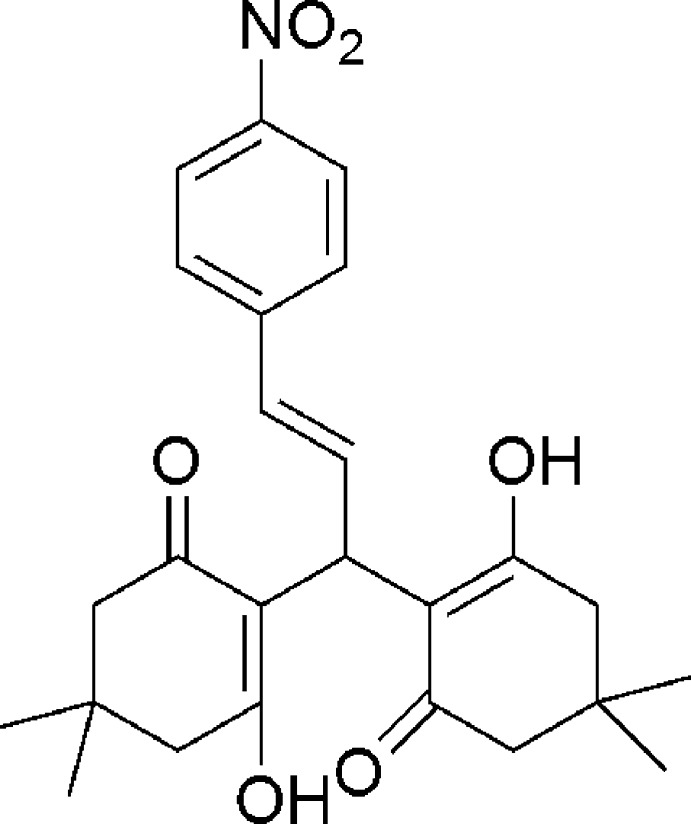


## Experimental

### 

#### Crystal data

C_25_H_29_NO_6_
*M* *_r_* = 439.51
Monoclinic, 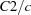

*a* = 25.0742 (13) Å
*b* = 10.2759 (5) Å
*c* = 20.7156 (9) Å
β = 119.7359 (13)°
*V* = 4634.7 (4) Å^3^
*Z* = 8
Mo *K*α radiation
μ = 0.09 mm^−1^
*T* = 296 K
0.30 × 0.10 × 0.10 mm

#### Data collection

Rigaku R-AXIS RAPID diffractometer
Absorption correction: multi-scan (*ABSCOR*; Rigaku, 1995 ▶) *T*_min_ = 0.792, *T*_max_ = 0.991
22032 measured reflections
5269 independent reflections
2991 reflections with *F* ^2^ > 2σ(*F* ^2^)
*R*_int_ = 0.032

#### Refinement

*R*[*F* ^2^ > 2σ(*F* ^2^)] = 0.042
*wR*(*F* ^2^) = 0.135
*S* = 1.06
5269 reflections
303 parameters
H-atom parameters constrained
Δρ_max_ = 0.18 e Å^−3^
Δρ_min_ = −0.22 e Å^−3^

### 

Data collection: *RAPID-AUTO* (Rigaku, 2006 ▶); cell refinement: *RAPID-AUTO*; data reduction: *RAPID-AUTO*; program(s) used to solve structure: *Il Milione* (Burla *et al.*, 2007 ▶); program(s) used to refine structure: *SHELXL97* (Sheldrick, 2008 ▶); molecular graphics: *CrystalStructure* (Rigaku, 2010 ▶); software used to prepare material for publication: *CrystalStructure*.

## Supplementary Material

Crystal structure: contains datablock(s) global, I. DOI: 10.1107/S1600536812032242/bh2444sup1.cif

Structure factors: contains datablock(s) I. DOI: 10.1107/S1600536812032242/bh2444Isup2.hkl

Supplementary material file. DOI: 10.1107/S1600536812032242/bh2444Isup3.cml

Additional supplementary materials:  crystallographic information; 3D view; checkCIF report

## Figures and Tables

**Table 1:** Hydrogen-bond geometry (Å, °)

*D*—H⋯*A*	*D*—H	H⋯*A*	*D*⋯*A*	*D*—H⋯*A*
O1—H1⋯O4	0.82	1.80	2.599 (2)	164
O2—H2⋯O3	0.82	1.87	2.658 (2)	162
C20—H20⋯O4^i^	0.93	2.39	3.248 (3)	154
C22—H22*C*⋯O5^ii^	0.96	2.58	3.438 (3)	148

Symmetry codes: (i) 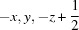; (ii) 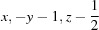.

**Fig. 1.**
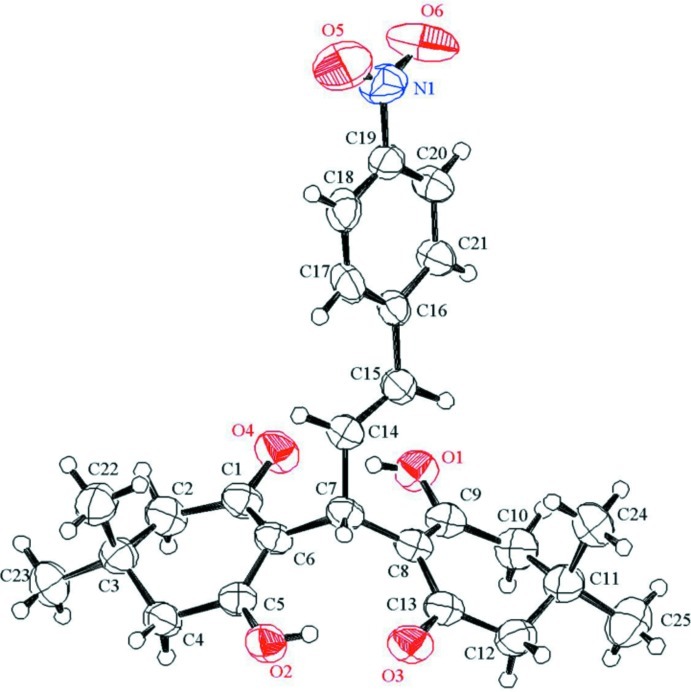
The molecular structure of the title molecule showing 50% probability displacement ellipsoids.

## supplementary crystallographic information

### Comment

As part of our ongoing study of the substituent effect on the solid state structures of two cyclohexenone ring derivatives (Cha *et al.*, 2011, 2012), we present here the crystal structure of the title compound (Fig. 1). The bond lengths and angles are normal and correspond to those observed in related structures (Cha *et al.*, 2011, 2012; Zhu *et al.*, 2011). Both cyclohexenone rings display half-chair conformations. The hydroxy and carbonyl O atoms face each other and are oriented to allow for the formation of two intramolecular O—H···O hydrogen bonds (Table 1). In the crystal, weak intermolecular C—H···O hydrogen bonds (Table 1) are formed between molecules, generating a 2D supramolecular structure.

### Experimental

To a solution of 5,5-dimethyl-1,3-cyclohexanedione (4.61 mmol), 4-nitrocinnamaldehyde (1.84 mmol) with 4 Å MS, was added small amounts of *L*-proline (0.47 mmol) under nitrogen atmosphere. Anhydrous ethyl acetate (2 ml) was added to the reaction mixture, and the solution was stirred for 1 day. The reaction mixture was filtered through a pad of celite in order to remove MS, and evaporation of the solvent afforded a mixture. The mixture was purified by flash column chromatography to afford the title product, which was recrystallized from ethanol, to give crystals suitable for X-ray analysis.

### Refinement

All C-bonded H atoms were positioned geometrically and refined using a riding model, with C—H = 0.93–0.98 Å. Hydroxyl H atoms, H1 and H2, were found in a difference map. Their positions were however fixed in an ideal geometry in the last refinement cycles, with O—H = 0.82 Å. For all H atoms, isotropic displacement parameters were computed as *U*_iso_(H) = 1.2*U*_eq_(carrier atom).

### Crystal data

**Table:**

C_25_H_29_NO_6_	*F*(000) = 1872.00
*M**_r_* = 439.51	*D*_x_ = 1.260 Mg m^−^^3^
Monoclinic, *C*2/*c*	Mo *K*α radiation, λ = 0.71075 Å
Hall symbol: -C 2yc	Cell parameters from 13620 reflections
*a* = 25.0742 (13) Å	θ = 3.2–27.5°
*b* = 10.2759 (5) Å	µ = 0.09 mm^−^^1^
*c* = 20.7156 (9) Å	*T* = 296 K
β = 119.7359 (13)°	Block, colourless
*V* = 4634.7 (4) Å^3^	0.30 × 0.10 × 0.10 mm
*Z* = 8	

### Data collection

**Table:**

Rigaku R-AXIS RAPID diffractometer	2991 reflections with *F*^2^ > 2σ(*F*^2^)
Detector resolution: 10.000 pixels mm^-1^	*R*_int_ = 0.032
ω scans	θ_max_ = 27.5°
Absorption correction: multi-scan (*ABSCOR*; Rigaku, 1995)	*h* = −32→32
*T*_min_ = 0.792, *T*_max_ = 0.991	*k* = −13→13
22032 measured reflections	*l* = −26→26
5269 independent reflections	

### Refinement

**Table:**

Refinement on *F*^2^	Primary atom site location: structure-invariant direct methods
*R*[*F*^2^ > 2σ(*F*^2^)] = 0.042	Secondary atom site location: difference Fourier map
*wR*(*F*^2^) = 0.135	Hydrogen site location: inferred from neighbouring sites
*S* = 1.06	H-atom parameters constrained
5269 reflections	*w* = 1/[σ^2^(*F*_o_^2^) + (0.0717*P*)^2^] where *P* = (*F*_o_^2^ + 2*F*_c_^2^)/3
303 parameters	(Δ/σ)_max_ < 0.001
0 restraints	Δρ_max_ = 0.18 e Å^−^^3^
0 constraints	Δρ_min_ = −0.22 e Å^−^^3^

### Fractional atomic coordinates and isotropic or equivalent isotropic displacement parameters (Å^2^)

**Table:**

	*x*	*y*	*z*	*U*_iso_*/*U*_eq_	
O1	0.02930 (5)	0.13724 (12)	0.14367 (7)	0.0573 (4)	
O2	0.23893 (5)	0.11823 (12)	0.13709 (7)	0.0589 (4)	
O3	0.21928 (5)	0.31987 (12)	0.20295 (6)	0.0603 (4)	
O4	0.05837 (6)	−0.07857 (13)	0.10323 (7)	0.0643 (4)	
O5	0.15275 (12)	−0.42718 (19)	0.52346 (12)	0.1368 (9)	
O6	0.06819 (10)	−0.3408 (3)	0.50333 (10)	0.1206 (8)	
N1	0.11093 (11)	−0.34889 (18)	0.49193 (9)	0.0768 (6)	
C1	0.10440 (7)	−0.07296 (17)	0.09272 (8)	0.0491 (4)	
C2	0.10272 (8)	−0.16048 (19)	0.03373 (9)	0.0578 (5)	
C3	0.16465 (8)	−0.18324 (17)	0.03870 (8)	0.0513 (5)	
C4	0.19570 (8)	−0.05109 (17)	0.04924 (9)	0.0512 (4)	
C5	0.19625 (7)	0.02967 (16)	0.10971 (8)	0.0446 (4)	
C6	0.15405 (7)	0.01186 (15)	0.13362 (8)	0.0429 (4)	
C7	0.16143 (7)	0.08327 (15)	0.20220 (7)	0.0422 (4)	
C8	0.13044 (7)	0.21521 (15)	0.18834 (7)	0.0422 (4)	
C9	0.06831 (7)	0.23269 (16)	0.16367 (8)	0.0476 (4)	
C10	0.04089 (8)	0.36434 (18)	0.15737 (10)	0.0597 (5)	
C11	0.08671 (9)	0.46759 (18)	0.20644 (9)	0.0571 (5)	
C12	0.14051 (9)	0.46117 (17)	0.19166 (10)	0.0582 (5)	
C13	0.16543 (8)	0.32739 (16)	0.19520 (8)	0.0474 (4)	
C14	0.15201 (7)	−0.00807 (17)	0.25298 (8)	0.0458 (4)	
C15	0.12608 (8)	0.01416 (17)	0.29326 (8)	0.0478 (4)	
C16	0.12309 (7)	−0.08092 (15)	0.34496 (7)	0.0429 (4)	
C17	0.16846 (8)	−0.17428 (17)	0.38157 (9)	0.0536 (5)	
C18	0.16503 (9)	−0.26281 (17)	0.42933 (9)	0.0566 (5)	
C19	0.11526 (8)	−0.25652 (16)	0.44083 (8)	0.0503 (5)	
C20	0.06988 (8)	−0.16539 (17)	0.40619 (9)	0.0536 (5)	
C21	0.07438 (8)	−0.07802 (17)	0.35877 (8)	0.0502 (4)	
C22	0.20484 (10)	−0.27305 (19)	0.10411 (10)	0.0673 (6)	
C23	0.15459 (10)	−0.2466 (2)	−0.03369 (10)	0.0694 (6)	
C24	0.10892 (10)	0.4410 (2)	0.28900 (10)	0.0713 (6)	
C25	0.05730 (12)	0.6024 (2)	0.18634 (14)	0.0861 (7)	
H1	0.0449	0.0712	0.1380	0.0688*	
H2A	0.0749	−0.1232	−0.0145	0.0694*	
H2B	0.0860	−0.2440	0.0366	0.0694*	
H2	0.2324	0.1681	0.1633	0.0707*	
H4A	0.2377	−0.0648	0.0605	0.0615*	
H4B	0.1747	−0.0032	0.0029	0.0615*	
H7	0.2053	0.1039	0.2303	0.0506*	
H10A	0.0087	0.3576	0.1703	0.0716*	
H10B	0.0220	0.3926	0.1060	0.0716*	
H12A	0.1274	0.4972	0.1428	0.0698*	
H12B	0.1734	0.5157	0.2277	0.0698*	
H14	0.1691	−0.0914	0.2554	0.0635*	
H15	0.1059	0.0960	0.2895	0.0615*	
H17	0.2019	−0.1770	0.3736	0.0643*	
H18	0.1955	−0.3253	0.4533	0.0680*	
H20	0.0367	−0.1626	0.4145	0.0643*	
H21	0.0439	−0.0154	0.3354	0.0603*	
H22A	0.2102	−0.2355	0.1494	0.0808*	
H22B	0.2442	−0.2835	0.1075	0.0808*	
H22C	0.1853	−0.3564	0.0964	0.0808*	
H23A	0.1935	−0.2582	−0.0314	0.0833*	
H23B	0.1289	−0.1914	−0.0750	0.0833*	
H23C	0.1351	−0.3296	−0.0399	0.0833*	
H24A	0.1293	0.3581	0.3027	0.0856*	
H24B	0.0743	0.4401	0.2970	0.0856*	
H24C	0.1370	0.5081	0.3188	0.0856*	
H25A	0.0422	0.6188	0.1345	0.1034*	
H25B	0.0873	0.6670	0.2155	0.1034*	
H25C	0.0239	0.6062	0.1964	0.1034*	

### Atomic displacement parameters (Å^2^)

**Table:**

	*U*^11^	*U*^22^	*U*^33^	*U*^12^	*U*^13^	*U*^23^
O1	0.0389 (7)	0.0618 (8)	0.0668 (7)	−0.0098 (6)	0.0228 (6)	−0.0031 (7)
O2	0.0524 (7)	0.0634 (8)	0.0726 (8)	−0.0149 (6)	0.0397 (6)	−0.0106 (6)
O3	0.0472 (8)	0.0684 (8)	0.0680 (8)	−0.0200 (6)	0.0306 (6)	−0.0070 (6)
O4	0.0494 (7)	0.0801 (9)	0.0723 (8)	−0.0236 (7)	0.0370 (6)	−0.0191 (7)
O5	0.213 (3)	0.0915 (14)	0.1371 (16)	0.0556 (15)	0.1105 (17)	0.0667 (13)
O6	0.1199 (16)	0.1614 (19)	0.0983 (12)	−0.0118 (14)	0.0677 (12)	0.0483 (12)
N1	0.1114 (16)	0.0632 (11)	0.0575 (10)	−0.0026 (11)	0.0432 (11)	0.0125 (9)
C1	0.0442 (10)	0.0600 (11)	0.0435 (9)	−0.0092 (8)	0.0220 (7)	−0.0014 (8)
C2	0.0568 (11)	0.0677 (12)	0.0465 (9)	−0.0152 (9)	0.0238 (8)	−0.0105 (9)
C3	0.0588 (11)	0.0542 (10)	0.0410 (8)	−0.0016 (9)	0.0247 (8)	0.0001 (8)
C4	0.0555 (11)	0.0569 (11)	0.0507 (9)	0.0006 (9)	0.0335 (8)	0.0045 (8)
C5	0.0408 (9)	0.0482 (9)	0.0448 (8)	−0.0044 (8)	0.0213 (7)	0.0032 (7)
C6	0.0377 (9)	0.0525 (10)	0.0386 (8)	−0.0072 (7)	0.0191 (7)	−0.0003 (7)
C7	0.0350 (8)	0.0535 (10)	0.0386 (8)	−0.0079 (7)	0.0187 (6)	−0.0014 (7)
C8	0.0372 (9)	0.0527 (10)	0.0350 (8)	−0.0070 (7)	0.0166 (7)	−0.0004 (7)
C9	0.0422 (9)	0.0573 (10)	0.0409 (8)	−0.0061 (8)	0.0187 (7)	0.0024 (8)
C10	0.0501 (11)	0.0646 (12)	0.0596 (10)	0.0049 (9)	0.0237 (9)	0.0064 (9)
C11	0.0635 (12)	0.0546 (11)	0.0538 (10)	0.0007 (9)	0.0296 (9)	0.0021 (8)
C12	0.0665 (12)	0.0533 (11)	0.0551 (10)	−0.0117 (9)	0.0305 (9)	−0.0021 (8)
C13	0.0474 (10)	0.0560 (11)	0.0388 (8)	−0.0128 (8)	0.0213 (7)	−0.0040 (7)
C14	0.0463 (10)	0.0507 (10)	0.0422 (8)	0.0000 (8)	0.0234 (7)	0.0017 (7)
C15	0.0520 (10)	0.0490 (10)	0.0479 (9)	0.0046 (8)	0.0289 (8)	0.0032 (8)
C16	0.0466 (9)	0.0473 (9)	0.0380 (8)	0.0043 (8)	0.0233 (7)	0.0001 (7)
C17	0.0537 (10)	0.0630 (11)	0.0550 (9)	0.0118 (9)	0.0352 (8)	0.0002 (9)
C18	0.0657 (12)	0.0516 (10)	0.0509 (10)	0.0215 (9)	0.0275 (9)	0.0073 (8)
C19	0.0658 (12)	0.0455 (9)	0.0414 (9)	−0.0016 (9)	0.0280 (8)	0.0019 (7)
C20	0.0482 (10)	0.0657 (12)	0.0531 (9)	0.0010 (9)	0.0299 (8)	0.0060 (9)
C21	0.0471 (10)	0.0577 (10)	0.0502 (9)	0.0113 (8)	0.0274 (8)	0.0117 (8)
C22	0.0855 (15)	0.0578 (12)	0.0558 (11)	0.0031 (10)	0.0329 (10)	0.0036 (9)
C23	0.0862 (15)	0.0716 (13)	0.0548 (11)	−0.0024 (11)	0.0384 (10)	−0.0098 (9)
C24	0.0857 (15)	0.0758 (14)	0.0627 (11)	0.0009 (12)	0.0445 (11)	−0.0050 (10)
C25	0.0941 (17)	0.0654 (14)	0.0990 (16)	0.0138 (12)	0.0481 (14)	0.0090 (12)

### Geometric parameters (Å, º)

**Table:**

O1—C9	1.299 (2)	C19—C20	1.370 (3)
O2—C5	1.301 (2)	C20—C21	1.376 (3)
O3—C13	1.281 (3)	O1—H1	0.820
O4—C1	1.278 (3)	O2—H2	0.820
O5—N1	1.222 (3)	C2—H2A	0.970
O6—N1	1.209 (4)	C2—H2B	0.970
N1—C19	1.465 (3)	C4—H4A	0.970
C1—C2	1.501 (3)	C4—H4B	0.970
C1—C6	1.407 (2)	C7—H7	0.980
C2—C3	1.523 (3)	C10—H10A	0.970
C3—C4	1.526 (3)	C10—H10B	0.970
C3—C22	1.533 (3)	C12—H12A	0.970
C3—C23	1.536 (3)	C12—H12B	0.970
C4—C5	1.497 (3)	C14—H14	0.947
C5—C6	1.385 (3)	C15—H15	0.965
C6—C7	1.527 (3)	C17—H17	0.930
C7—C8	1.518 (3)	C18—H18	0.930
C7—C14	1.513 (3)	C20—H20	0.930
C8—C9	1.389 (3)	C21—H21	0.930
C8—C13	1.412 (3)	C22—H22A	0.960
C9—C10	1.494 (3)	C22—H22B	0.960
C10—C11	1.523 (3)	C22—H22C	0.960
C11—C12	1.526 (4)	C23—H23A	0.960
C11—C24	1.538 (3)	C23—H23B	0.960
C11—C25	1.527 (3)	C23—H23C	0.960
C12—C13	1.497 (3)	C24—H24A	0.960
C14—C15	1.307 (3)	C24—H24B	0.960
C15—C16	1.479 (3)	C24—H24C	0.960
C16—C17	1.391 (3)	C25—H25A	0.960
C16—C21	1.386 (3)	C25—H25B	0.960
C17—C18	1.378 (3)	C25—H25C	0.960
C18—C19	1.383 (4)		
			
O5—N1—O6	123.3 (3)	C3—C2—H2B	108.542
O5—N1—C19	117.5 (3)	H2A—C2—H2B	107.531
O6—N1—C19	119.2 (2)	C3—C4—H4A	108.773
O4—C1—C2	116.67 (15)	C3—C4—H4B	108.780
O4—C1—C6	121.97 (17)	C5—C4—H4A	108.765
C2—C1—C6	121.36 (18)	C5—C4—H4B	108.768
C1—C2—C3	114.91 (13)	H4A—C4—H4B	107.669
C2—C3—C4	107.69 (16)	C6—C7—H7	103.784
C2—C3—C22	110.71 (18)	C8—C7—H7	103.783
C2—C3—C23	109.32 (14)	C14—C7—H7	103.786
C4—C3—C22	110.14 (13)	C9—C10—H10A	108.695
C4—C3—C23	109.97 (18)	C9—C10—H10B	108.693
C22—C3—C23	109.00 (16)	C11—C10—H10A	108.702
C3—C4—C5	113.91 (18)	C11—C10—H10B	108.695
O2—C5—C4	114.63 (18)	H10A—C10—H10B	107.630
O2—C5—C6	123.06 (17)	C11—C12—H12A	108.576
C4—C5—C6	122.30 (15)	C11—C12—H12B	108.569
C1—C6—C5	118.10 (16)	C13—C12—H12A	108.568
C1—C6—C7	121.38 (18)	C13—C12—H12B	108.570
C5—C6—C7	120.52 (14)	H12A—C12—H12B	107.553
C6—C7—C8	116.04 (11)	C7—C14—H14	112.040
C6—C7—C14	111.11 (14)	C15—C14—H14	118.601
C8—C7—C14	116.35 (17)	C14—C15—H15	120.557
C7—C8—C9	124.09 (15)	C16—C15—H15	114.895
C7—C8—C13	118.42 (16)	C16—C17—H17	119.287
C9—C8—C13	117.30 (15)	C18—C17—H17	119.274
O1—C9—C8	123.30 (16)	C17—C18—H18	120.800
O1—C9—C10	114.50 (15)	C19—C18—H18	120.787
C8—C9—C10	122.19 (15)	C19—C20—H20	120.693
C9—C10—C11	114.23 (14)	C21—C20—H20	120.686
C10—C11—C12	106.57 (17)	C16—C21—H21	119.148
C10—C11—C24	110.82 (18)	C20—C21—H21	119.119
C10—C11—C25	110.34 (15)	C3—C22—H22A	109.474
C12—C11—C24	110.30 (16)	C3—C22—H22B	109.469
C12—C11—C25	109.9 (2)	C3—C22—H22C	109.474
C24—C11—C25	108.9 (2)	H22A—C22—H22B	109.479
C11—C12—C13	114.78 (18)	H22A—C22—H22C	109.464
O3—C13—C8	121.81 (16)	H22B—C22—H22C	109.468
O3—C13—C12	116.75 (17)	C3—C23—H23A	109.477
C8—C13—C12	121.42 (19)	C3—C23—H23B	109.471
C7—C14—C15	129.33 (17)	C3—C23—H23C	109.481
C14—C15—C16	124.52 (17)	H23A—C23—H23B	109.471
C15—C16—C17	121.95 (19)	H23A—C23—H23C	109.467
C15—C16—C21	120.11 (15)	H23B—C23—H23C	109.459
C17—C16—C21	117.94 (17)	C11—C24—H24A	109.465
C16—C17—C18	121.4 (2)	C11—C24—H24B	109.466
C17—C18—C19	118.41 (17)	C11—C24—H24C	109.468
N1—C19—C18	119.38 (17)	H24A—C24—H24B	109.472
N1—C19—C20	118.8 (2)	H24A—C24—H24C	109.473
C18—C19—C20	121.85 (18)	H24B—C24—H24C	109.484
C19—C20—C21	118.6 (2)	C11—C25—H25A	109.468
C16—C21—C20	121.73 (16)	C11—C25—H25B	109.480
C9—O1—H1	109.478	C11—C25—H25C	109.465
C5—O2—H2	109.479	H25A—C25—H25B	109.484
C1—C2—H2A	108.549	H25A—C25—H25C	109.463
C1—C2—H2B	108.544	H25B—C25—H25C	109.469
C3—C2—H2A	108.531		
			
O5—N1—C19—C18	1.2 (3)	C7—C8—C9—O1	−6.3 (3)
O5—N1—C19—C20	−178.30 (16)	C7—C8—C9—C10	174.32 (13)
O6—N1—C19—C18	178.05 (16)	C7—C8—C13—O3	9.3 (3)
O6—N1—C19—C20	−1.4 (3)	C7—C8—C13—C12	−172.36 (12)
O4—C1—C2—C3	−161.78 (13)	C9—C8—C13—O3	−165.86 (14)
O4—C1—C6—C5	−168.20 (13)	C9—C8—C13—C12	12.4 (3)
O4—C1—C6—C7	11.5 (3)	C13—C8—C9—O1	168.61 (15)
C2—C1—C6—C5	11.1 (2)	C13—C8—C9—C10	−10.8 (3)
C2—C1—C6—C7	−169.26 (13)	O1—C9—C10—C11	157.68 (16)
C6—C1—C2—C3	18.9 (3)	C8—C9—C10—C11	−22.9 (3)
C1—C2—C3—C4	−47.49 (18)	C9—C10—C11—C12	50.7 (2)
C1—C2—C3—C22	72.96 (18)	C9—C10—C11—C24	−69.3 (3)
C1—C2—C3—C23	−166.95 (14)	C9—C10—C11—C25	169.98 (17)
C2—C3—C4—C5	49.48 (15)	C10—C11—C12—C13	−49.23 (17)
C22—C3—C4—C5	−71.3 (2)	C24—C11—C12—C13	71.13 (18)
C23—C3—C4—C5	168.51 (13)	C25—C11—C12—C13	−168.79 (13)
C3—C4—C5—O2	157.31 (12)	C11—C12—C13—O3	−162.00 (13)
C3—C4—C5—C6	−23.43 (19)	C11—C12—C13—C8	19.6 (2)
O2—C5—C6—C1	170.53 (12)	C7—C14—C15—C16	−175.75 (11)
O2—C5—C6—C7	−9.1 (2)	C14—C15—C16—C17	30.3 (2)
C4—C5—C6—C1	−8.7 (2)	C14—C15—C16—C21	−150.32 (14)
C4—C5—C6—C7	171.66 (12)	C15—C16—C17—C18	−179.66 (12)
C1—C6—C7—C8	−89.53 (18)	C15—C16—C21—C20	179.62 (12)
C1—C6—C7—C14	46.37 (17)	C17—C16—C21—C20	−1.0 (2)
C5—C6—C7—C8	90.12 (17)	C21—C16—C17—C18	1.0 (2)
C5—C6—C7—C14	−133.98 (14)	C16—C17—C18—C19	−0.5 (3)
C6—C7—C8—C9	80.5 (2)	C17—C18—C19—N1	−179.43 (13)
C6—C7—C8—C13	−94.32 (15)	C17—C18—C19—C20	0.0 (3)
C6—C7—C14—C15	−143.57 (14)	N1—C19—C20—C21	179.40 (13)
C8—C7—C14—C15	−7.82 (19)	C18—C19—C20—C21	−0.1 (3)
C14—C7—C8—C9	−53.05 (17)	C19—C20—C21—C16	0.5 (3)
C14—C7—C8—C13	132.10 (13)		

### Hydrogen-bond geometry (Å, º)

**Table:**

*D*—H···*A*	*D*—H	H···*A*	*D*···*A*	*D*—H···*A*
O1—H1···O4	0.82	1.80	2.599 (2)	164
O2—H2···O3	0.82	1.87	2.658 (2)	162
C20—H20···O4^i^	0.93	2.39	3.248 (3)	154
C22—H22*C*···O5^ii^	0.96	2.58	3.438 (3)	148

Symmetry codes: (i) −*x*, *y*, −*z*+1/2; (ii) *x*, −*y*−1, *z*−1/2.
